# Hydroxyapatite Biobased Materials for Treatment and Diagnosis of Cancer 

**DOI:** 10.3390/ijms231911352

**Published:** 2022-09-26

**Authors:** María del Carmen De Lama-Odría, Luis J. del Valle, Jordi Puiggalí

**Affiliations:** 1 Departament d’Enginyeria Química, Universitat Politècnica de Catalunya, EEBE, Av. Eduard Maristany 10-14, E-08019 Barcelona, Spain; 2 Barcelona Research Center in Multiscale Science and Engineering, Universitat Politècnica de Catalunya, Campus Diagonal-Besòs, Av. Eduard Maristany 10-14, E-08019 Barcelona, Spain; 3 Institute for Bioengineering of Catalonia (IBEC), The Barcelona Institute of Science and Technology (BIST), Carrer Baldiri i Reixac 11-15, E-08028 Barcelona, Spain

**Keywords:** cancer, hydroxyapatite, drug-carrier, antitumoral, cell imaging, hyperthermia, immunotherapy, gene delivery

## Abstract

Great advances in cancer treatment have been undertaken in the last years as a consequence of the development of new antitumoral drugs able to target cancer cells with decreasing side effects and a better understanding of the behavior of neoplastic cells during invasion and metastasis. Specifically, drug delivery systems (DDS) based on the use of hydroxyapatite nanoparticles (HAp NPs) are gaining attention and merit a comprehensive review focused on their potential applications. These are derived from the intrinsic properties of HAp (e.g., biocompatibility and biodegradability), together with the easy functionalization and easy control of porosity, crystallinity and morphology of HAp NPs. The capacity to tailor the properties of DLS based on HAp NPs has well-recognized advantages for the control of both drug loading and release. Furthermore, the functionalization of NPs allows a targeted uptake in tumoral cells while their rapid elimination by the reticuloendothelial system (RES) can be avoided. Advances in HAp NPs involve not only their use as drug nanocarriers but also their employment as nanosystems for magnetic hyperthermia therapy, gene delivery systems, adjuvants for cancer immunotherapy and nanoparticles for cell imaging.

## 1. Introduction

Despite the progress made in prevention, diagnosis and treatment, cancer remains one of the most common causes of mortality worldwide and even represents the leading cause of death over cardiovascular diseases in several high-income countries [1,2]. The cancer burden is expected to keep rising, with a projection of a 61.7% increase in incidence worldwide by the year 2040, according to GLOBOCAN (World Health Organization’s International Agency for Research on Cancer Global Cancer Observatory) data [3].

A key point to understanding the current state of cancer-associated mortality and morbidity is that, albeit the advances in cancer biology, the new concepts of neoplastic cell behavior during invasion and metastasis have not been translated into the development of more effective treatments [1]. Insights into the complexity of carcinogenesis emphasize the need to devise new antitumoral treatments that can target the cancerous cells while decreasing side effects [2].

In this scenario, hydroxyapatite (HAp) has emerged as a biocompatible option for the design of targeted anticancer drug delivery systems (DDS) [2]. As the main inorganic component of mineralized tissues, HAp has a good biological activity with the facility to incorporate different molecules during the synthesis process and the advantage of tunable morphology, crystal size or crystallinity [4,5]. Moreover, the use of different removable templates during HAp synthesis allows the formation of porous structures (mesoporous HAp), characterized by increased surface area and permeability. The variable pore volume, pore size and distribution can improve drug loading, drug release and biocompatibility [6,7].

When compared to other inorganic systems for drug delivery, such as silica, quantum dots, carbon nanotubes or magnetic particles, HAp is less cytotoxic. Additionally, it has some advantages over organic systems. As an example, when compared to liposomes, it is not subject to dissipation below a critical concentration and it is not soluble in blood, translating into higher stability for intravenous administration [5]. Furthermore, the development of new gels based on HAp NPs and the self-assembly of peptides is attracting great attention [8].

Recent investigations also suggest a prohibitory effect of HAp on the metabolic activity of different tumoral cells with nuclei internalization, mitochondria internalization and apoptosis activation via alteration of mitochondrial membrane potential as possible mechanisms of antitumoral action [9,10,11,12,13]. 

Considering the aforementioned, we present a review of the progress in the field of HAp-based biomaterials for the treatment and diagnosis of cancer. For this purpose, the review is structured into different chapters that try to provide the following: (a) The fundamentals of glucose, glutamine and lipid metabolisms in cancer cells; (b) Basic concepts concerning cancer cell uptake of HAp as follows: Endocytosis, disruption of mitochondrial homeostasis and apoptosis, (c) Synthesis of HAp NPs, including tunability of properties such as crystallinity and morphology; (d) Considerations of HAp as an efficient antitumoral nanocarrier, including the potential given by doping and structural modification; (e) Functionalization of HAp NPs and inclusion on biopolymer matrices; (f) supermagnetic NPs based on HAp for cancer treatment; (g) Applications of HAp NPs in immunotherapy and gene delivery systems; (h) HAp NPs for imaging and cancer detection. Similarly, we highlight the need to provide a better understanding of the biological behavior of those systems *in vivo*.

## 2. Genetical Fundamentals and Metabolic Reprogramming in Cancer: Fundamentals for Therapeutic Target Selection 

Cancer can be defined as a multifactorial and multi-step disease [1]. From a genetic aspect, a sequence of reported mutations, epigenetic changes and genomic alterations are associated with the deregulation of cell replication, division and growth [1]. Even some oncogenes and tumor suppressor genes are linked to the alteration of metabolic pathways (such as the aerobic glycolytic pathway or the glutamine catabolic pathway) and the acquisition of mechanisms for drug resistance [14]. Nevertheless, not all mutational events are common to all types of cancer, with some subsets being associated with specific genotypes [15]. As an example, key alterations of APC (adenomatous polyposis coli), TP53 (tumor protein p53), KRAS (Kirsten rat sarcoma virus) and SMAD4 (SMAD family member 4) genes that drive colon cancer development have not been shown to be responsible for the progression of other cancers [16].

As aforementioned, genetic alterations can facilitate the fast and large generation of macromolecules and metabolic intermediates that are highly demanded by neoplastic cells for growth and sustained proliferation [14]. Progress in metabolomics has remarked the importance of metabolic reprogramming in cancer cells’ survival and metastasis, introducing the term oncometabolites to describe every endogenous metabolite that accumulates in the tumor (i.e., fumarate in renal cell carcinoma or choline in prostate and brain cancer) [14,17,18]. Part of the pathological phenotype of cancer cells is the defective mitochondrial oxidative phosphorylation that leads to the activation of aerobic fermentation of lactic acid. It also activates the mitochondrial substrate-level phosphorylation, which leads to enhanced energy production and the collateral synthesis of reactive oxygen species (ROS) that have mutagenic and carcinogenic potential [19,20]. Recent findings suggest that the dependency of cancer cells in the glycolytic pathway relies not only on the adenosine triphosphate (ATP) production but on the synthesis of glycolytic intermediates that are basic precursors for anabolic pathways [1].

**Figure 1:**
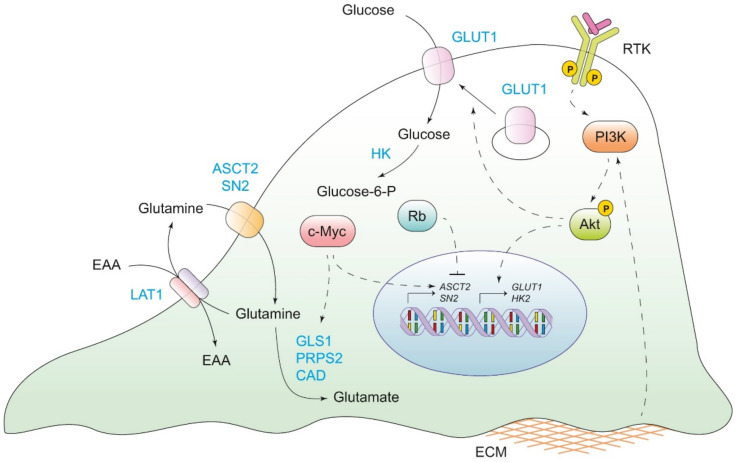
Deregulation of glucose and amino acids uptake in cancer cells. Solid arrows represent the movement of metabolites and proteins or the metabolic reactions. The dashed arrows indicate either positive or negative regulatory effects on signaling pathways in cancer cells. RTK: receptor tyrosine kinase; GLUT1: glucose transporter 1; ASCT2/SN2: glutamine transporter; LAT1: neutral amino acid transporter; EAA: essential amino acids; GLS1: glutaminase 1; PRPS2: phosphoribosyl pyrophosphate synthetase 2; CAD: carbamoyl-phosphate synthetase 2; HK: hexokinase; ECM: extracellular matrix. Image reproduced from [22] with permission from Elsevier. Copyright © 2016.

An important step for metabolic reprogramming is the increased expression of enzymes that participate in glucose metabolism. For example, the activation of the PI3K/Akt signaling pathway increases the expression and translocation of GLUT1 and potentiates the activity of the hexokinase for glucose phosphorylation. Similarly, the transcription factor c-Myc, a downstream molecule in the PI3K/Akt pathway, is overexpressed in tumoral cells and can activate target genes for glucose transport and lactate efflux (Figure 1) [21,22,23,24]. As oxygen supply to the cancer cells is low in the tumoral microenvironment due to the blood vessel quality, hypoxia-inducible factors 1 and 2 (transcription factors) can also be constitutively activated to regulate the expression of glucose transporters and glycolytic enzymes (i.e., GLUT1, GLUT3 and HK2) [1,25].

**Figure 2:**
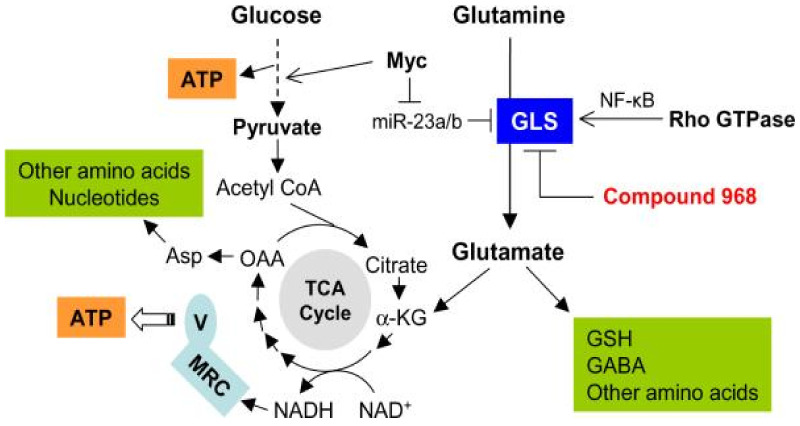
Regulation of glutamine metabolism in cancer cell through the modulation of glutaminase. As depicted in the figure, glucose and glutamine metabolism are interconnected. Assays performed with compound 968, an inhibitor of glutaminase, emphasized the importance of glutamine in the biochemistry of cancer cells. GLS: glutaminase; TCA cycle: tricarboxylic acid cycle; MRC: mitochondrial respiratory chain; V: mitochondrial respiratory complex V; OAA: oxaloacetate; Asp: aspartate; α-KG: α-ketoglutarate. Image reproduced from [26] with permission from Elsevier. Copyright © 2010.

**Figure 3:**
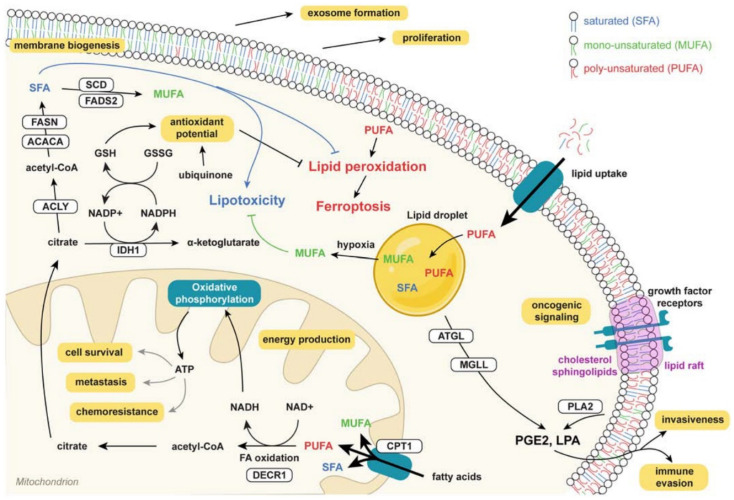
Role of lipids in cancer biology. Cancer cells are dependent on fatty acid synthesis for membrane biogenesis during cell proliferation. Their oxidation generates ATP and increases antioxidant potential through glutathione synthesis. Additionally, they participate in cell signaling mechanisms. To prevent the peroxidation of polyunsaturated fatty acids, tumoral cells store this type of lipids or catabolize them via Fas oxidation pathways. Image republished from [28] with permission from Elsevier. Copyright © 2020.

Another common metabolic feature is the increased levels of glutamine and lipid metabolism reprogramming. Glutamine is required for oxidative metabolism, ATP generation, redox homeostasis and as an anaplerotic substrate in the TCA cycle (Figure 2) [26,27]. In the case of lipids, de novo lipogenesis (DNL) and exogenous fatty acids (Fas) uptake support the membrane formation, energy storage, ATP production (via Fas oxidation) and synthesis of signaling molecules (Figure 3) [28]. As observed with glucose and glutamine, crucial regulators of lipid metabolism are upregulated in tumoral cells, such as the acetyl-CoA carboxylase (ACC), the fatty acid synthase (FASN) or the stearoyl-CoA desaturase 1 (SCD1) [29,30].

## 3. Mechanisms of Endocytosis, Disruption of Mitochondrial Ca^2+^ Homeostasis and Apoptosis Triggered by HAp Uptake in Cancer Cells

**Figure 4:**
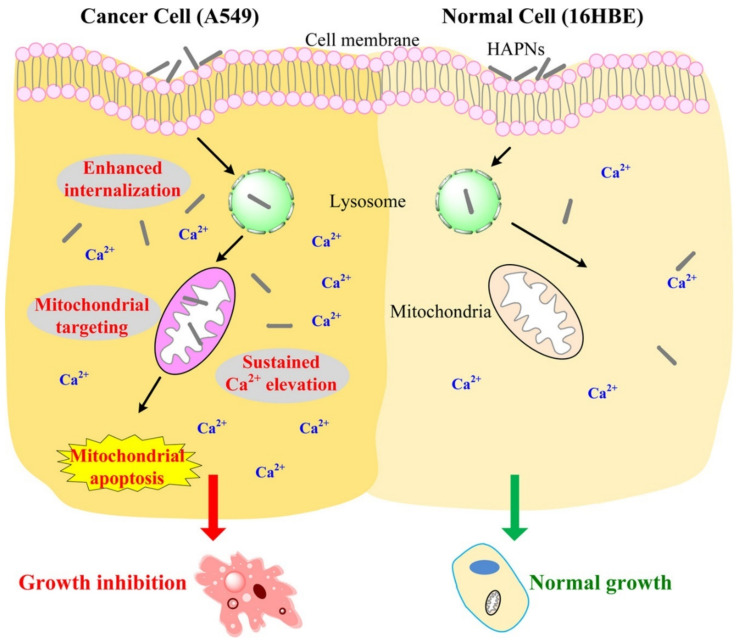
HAp-induced mitochondria-mediated apoptosis. Internalization of HAp NPs in A549 cells is represented. The intracellular Ca^2+^ overload can induce mitochondria damage and activation of apoptotic mechanisms. It is proposed that normal cells, as the bronchial epithelial cell line 16HBE depicted in this figure, can restore the homeostasis of this cation. Figure republished from [33] with permission from the American Chemical Society. Copyright © 2016.

HAp nanoparticles (NPs) are uptaken by cancer cells according to mechanisms that depend on different properties, such as morphology or crystal size. In the lysosomes, the HAp NPs are degraded and the constitutive Ca^2+^ ions are released into the cytosol. An intracellular imbalance of the cation concentrations ([Ca^2+^]_i_) will lead to the intake of the ion by the mitochondria in order to obtain homeostasis (normal cytosolic values are <200 nM and 400–600 μM in the lysosomes) [31,32]. However, excessive Ca^2+^ concentrations will activate cellular apoptosis. In a cancer cell, changes in Ca^2+^ transportation, the altered intracellular concentration of the cation and changes in the Ca^2+^-dependent signal pathways have been reported. On the other hand, the sensitivity of the tumoral cells to overloading of Ca^2+^ in the mitochondria has also been proposed (Figure 4) [31,33].

**Figure 5:**
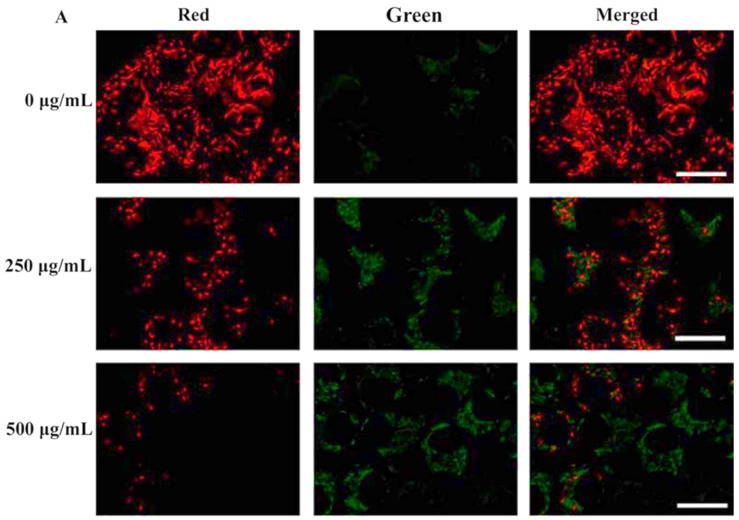
Assessment of mitochondrial membrane-potential in A549 after HAp-treatment. Confocal micrographs of JC-1-labeled A549 cells after 48 h of treatment with HAp NPs with rod-like morphology are shown. The red signal corresponds to the JC-1 aggregates that are formed in a mitochondrial membrane potential-dependent manner. The green signal corresponds to the JC-1 monomers emission. Scale bar: 20 µm. Figure republished from [33] with permission from the American Chemical Society. Copyright © 2016.

Tracking of [Ca^2+^]_i_ in normal cells and A549 lung cancer cells after 6 h of treatment with HAp NPs demonstrated that, while in the first group there was a decline in the concentration, in the neoplastic cells the concentration continued to increase, reaching the highest value at 24 h (1.5-fold higher than the control). Additionally, assessment of HAp NPs mobilization after internalization into the lung cancer cells revealed their translocation to the mitochondria accompanied by a reduction of the organelle membrane potential (suggested by the reduction of the red fluorescence signal of JC-1 polymers) (Figure 5) [33]. *In vitro* assays with melanoma cells showed vacuole formation in the nuclear membrane and shrinkage or fragmentation of the nucleus. The effect was observed only one day after being treated with 200 µg/mL of HAp NPs. After 3 days, the expression of BAX, BCL-2 and p53 genes was highly up-regulated, confirming the effect of HAp NPs in the tumoral cell line [34].

In another study with gastric cancer cells, hypodiploid DNA content and DNA fragmentation were reported after treatment with HAp NPs [35]. Similar results were obtained with lung cancer cells [33], osteosarcoma cells [36], breast cancer cells [37], colon cancer cells [38] and liver cancer cells [11,39].

*In vivo* studies were also performed with a rabbit intramuscular VX2 tumor model. It was found that the tumor size was 10 times smaller than the control after 3 weeks of treatment with HAp NPs. Histological staining of the tumor samples showed an invasion of the tumoral mass into adjacent muscle layers in the control groups, while a barrier of aggregate NPs was evidenced in the treatment group. Moreover, infiltration of immune cells, such as foreign body multinucleated giant cells, mononuclear cells, lymphocyte cells and neutrophil cells, was detected in the last group. Interestingly, expansion of the nuclear membrane, vacuolated mitochondria, aggregation of chromatin and formation of the apoptotic body were observed in the HAp NPs group, corroborating the *in vitro* reports [40]. The HAp NPs inhibited tumor growth with a 40% reduction of the tumoral mass and without general toxicity, as reported in another *in vivo* study performed with the BALB/c nu/nu nude mouse lung cancer model [33].

In the particular case of gliomas, studies performed with the human glioma U87MG ATCC and rat glioma C6 cells revealed that HAp NPs at a concentration of 20 µg/mL induced cell detachment after 24 h of treatment. Inhibition of invasion of human cells was demonstrated for concentrations ranging from 20 to 60 µg/mL. Thus, at a concentration above 25 µg/mL, reduced NF-κB p65 protein expression and blocked NF-κB p65 nuclear translocation was detected with C6 cells. Induction of apoptosis, cell cycle arrest at G2/M and decreased NF-κB target molecular expression were reported in both cell lines. Altogether, it was suggested that HAp NPs mediate cell death of glioma cells by the downregulation of NF-κB signaling [41].

Based on the exposed results, calcium phosphates are now considered for the design of DDS for cancer treatment in an attempt to induce a persistent influx of Ca^2+^ into the cytosol while transporting antitumoral pharmaceuticals [31].

## 4. Synthesis, Characteristics and Tailorable Physicochemical Properties of HAp for the Cancer Treatment

The crystalline structure of HAp is defined by a monoclinic *P2_1_/b* space group (*a* = 0.984 nm, *b* = 2*a*, *c* = 0.688 nm and *γ* = 120°) that converts at high temperature to a hexagonal *P6_3_/m* space group (*a* = *b* = 0.943 nm, *c* = 0.689 nm and *γ* = 120°) [42,43]. This structure is constituted by Ca^2+^ ions surrounded by tetrahedral PO_4_^3−^ units and OH^−^ ions, occupying columns that are parallel to the hexagonal axis. Ca^2+^ ions are mainly disposed at the *c*-surface and the PO_4_^3−^ and OH^−^ ions are located on the *a*-surface [44]. 

HAp nanoparticles are usually prepared by precipitation routes according to the following two main chemical reactions:

10 Ca(OH)_2_ + 6 H_3_PO_4_ → Ca_10_(PO_4_)_6_(OH)_2_ + 18 H_2_O (1)

10 Ca(NO_3_)_2_ + 6 (NH_4_)_2_HPO_4_ + 2 H_2_O → Ca_10_(PO_4_)_6_(OH)_2_ + 12 NH_4_NO_3_ + 8 HNO_3_ (2)

A great diversity of methods have been applied for HAp synthesis. They can be classified into (i) dry methods (e.g., solid state or mechanical route), (ii) wet methods (chemical precipitation, sol-gel, hydrothermal, emulsion and sonochemical routes), (iii) high-temperature processes (pyrolysis methods), (iv) methods based on biogenic sources, and (v) combinations of techniques [44,45,46,47].

An initial burst of nucleation that leads to a fast aggregation is characteristic of the precipitation process. The Van der Waal’s and secondary surface interactions between particles have a special relevancy to the observed aggregation [47]. To increase the bioavailability and internalization of the NPs by cancer cells, it is important to optimize the diameter range to 10–80 nm. Larger NPs can promptly accumulate in the reticuloendothelial system (RES), decreasing the blood circulation time. Conversely, NPs in the range of 8–10 nm are rapidly cleared by the renal system [48].

*In vitro* studies of the effect of morphology, size, specific surface area (SSA) and crystallinity on the viability of melanoma cells (A375 cell line) demonstrated that a higher antitumoral effect is achieved with a granular morphology, a smaller size, higher SSA and lower crystallinity values [34]. Similar results were obtained when treating human colon cancer HCT116 cells with HAp NPs, obtaining a higher cytotoxicity with rod-shaped morphology, a lower crystalline size (in a range of 11–22 nm) and crystallinity (average of 0.43) [38]. These results shed light on the variability in the antitumor effect of HAp that is a consequence of the combination of particle characteristics and not just one dominant trait [32].

**Figure 6:**
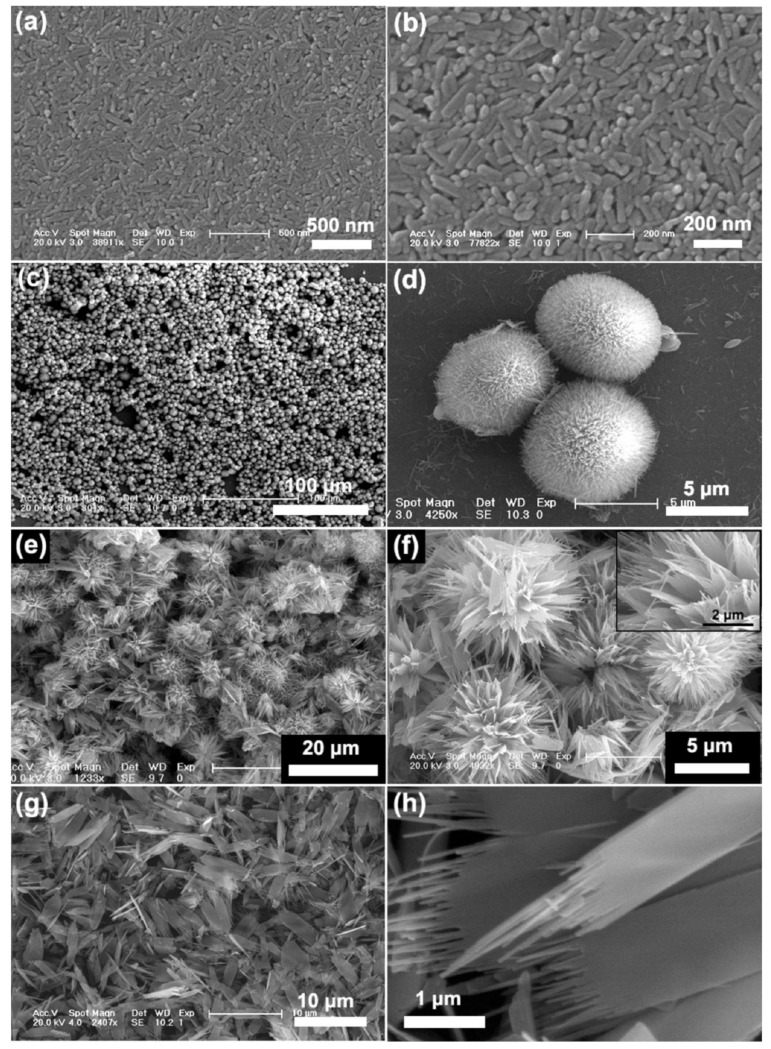
Nanorod (**a**,**b**), bur-like microsphere (**c**,**d**), microflower (**e**,**f**) and microsheet (**g**,**h**) morphologies that can be obtained from precipitation media having pH values of 7.0, 5.0, 4.5 and 4.0, respectively. Reproduced from [53] with permission from the American Chemical Society. Copyright © 2009.

Facile methods to control HAp NPs size include a variation of temperature, the concentration of calcium and phosphate ions to the mother solutions, way of the addition of reagents, pH, reaction time, the addition of chelating agents and presence of surfactants [49,50,51]. For example, the incorporation of magnesium or ferrous ions into the mineralization solution inhibits the crystal growth by distortion of the atomic structure of the NPs as smaller ions substitute the Ca^2+^ cations, leading to lattice contraction and reduced crystallinity. Especially in the case of iron in the trivalent form, which leads to the formation of only one Ca^2+^ vacancy per every two substitutions of the element by the ferric ion. These changes can be detected in the broadening of the diffraction peaks and the decreased (002)/(211) peak intensity ratio [48,52]. Figure 6 shows a clear example of the influence of pH conditions on the final morphology of HAp NPs [53]. The pore size of HAp NPs can be easily controlled by the selection of appropriate soft templates, co-surfactants and pore expanders [46].

The HAp surface area, surface charge (i.e., Ca/P ratio) and the charge of adsorbed molecules can influence the adsorption and release kinetics of the antitumoral drugs. In the particular case of cisplatin, a more negatively charged HAp surface favors the electrostatic binding of the cationic species of the hydrolyzed antitumoral agent. For this reason, HAp NPs morphology can condition the adsorption process by exposure to a higher (i.e., in plate-shaped HAp) or lower Ca/P ratio (i.e., in needle-shaped HAp) on the particle surface. With regard to cisplatin, the mentioned ratio should have lower values for increased adsorption. Drug desorption is not conditioned by the Ca/P ratio on the surface since only the surface area becomes a significant factor [54]. Barroug et al. also demonstrated that cisplatin absorption on the surface of calcium phosphate is temperature dependent and implies an endothermic process [55].

Surface modification of HAp NPs is relevant to establishing appropriate interactions with surrounding cells [56]. It is also important to favor interactions of HAp NPs with morphogenetic proteins, peptide growth factors and ionizable groups of active compounds in improving adsorption efficiency. Interactions may play a significant role to improve the compatibilization of HAp NPs with polymer matrices. The control of the roughness and charge of the surface, the pore size and the selection of the crystal growth face also have an important role in enhancing the indicated interactions [57,58]. Surface modification can involve a simple physical adsorption but also chemical immobilization (e.g., the establishment of covalent links, acid-base reactions and ionic bombardment). Inorganic phosphate derivatives such as bisphosphonates (BPs) and pyrophosphoric acid can be easily incorporated onto HAp surfaces. The first group is relevant due to its antiresorptive function and the capability to regulate the calcium metabolism. In any case, these derivatives are fundamental for their capability to bind basic proteins [59,60].

**Figure 7:**
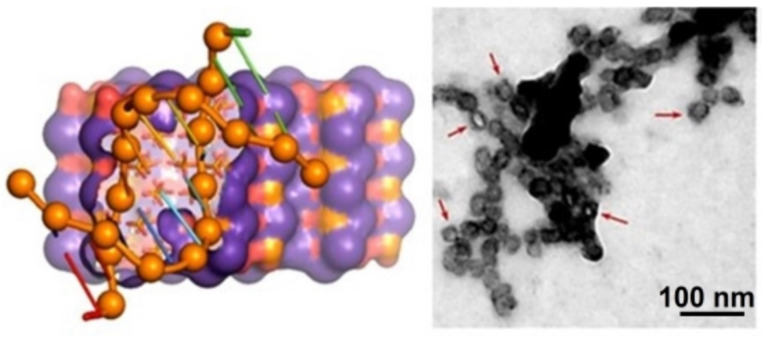
Scheme showing the incorporation of DNA inside the HAp structure (**left**) and TEM micrograph showing nanoparticles (red arrows) that incorporate DNA (**right**). Reproduced with permission from [64,65], respectively.

The HAp also has a high potential as a non-viral gene vector as a consequence of its practically null immunogenicity and the capacity to load transgenes with highly variable dimensions. Calcium ions of HAp can establish good interactions with the phosphate groups of DNA and consequently provide a capacity for the encapsulation and protection of DNA [61,62]. It has been indicated that HAp NPs can provide DNA protection, even against dNase digestion [63]. The capacity of HAp NPs to incorporate DNA with a minimum distortion of the crystalline structure has been postulated (Figure 7) [64,65].

## 5. Hydroxyapatite as an Antitumoral Drug Nanocarrier 

Cisplatin, a platinum (Pt) coordination compound, is used for the treatment of solid tumors. In detail, cisplatin has a square planar geometry with a central Pt atom that is coordinated by two chloride ions and two ammonia (NH_3_) ligands in the cis position. Hydrolysis of this molecule is normally suppressed in the extracellular media due to the high concentration of chloride ions. Once it is uptaken by the cell, the low cytosolic chloride ion concentration ([Cl^−^] is > 100 mM and close to 23 nM in extracellular plasma and cell cytoplasm, respectively) facilitates chlorine release and the formation of a reactive complex with water. This allows the establishment of covalent bonds with DNA that inhibit DNA synthesis and transcription. Additionally, the positively charged aquo-species, such as cis-[PtCl(NH_3_)_2_(H_2_O)]^+^, cis-[Pt(NH_3_)_2_(H_2_O)_2_]^2+^ and cis-[Pt(NH_3_)_2_(OH)(H_2_O)]^+^, can bind to nuclear and cytoplasmatic proteins, inducing a cytotoxic effect [66,67]. New generations of Pt compounds have been developed to reduce the side effects (e.g., oxaliplatin), evade the resistance to cisplatin by certain tumoral cells (e.g., oxaliplatin) or improve the solubility and water stability by incorporating a chelate pyrophosphate ligand (phosphaplatins) [68]. Nevertheless, the controlled delivery of these pharmaceutical agents is still a matter of interest in cancer therapy. In this context, nanocrystalline HAp has emerged as an alternative nanocarrier based on the demonstrated enhancement of drug release by the phosphate ions [68]. Moreover, investigations have shown an increased cisplatin adsorption and a slower drug release with a decreasing HAp crystallinity [54]. This has been attributed to the surface defects that create more active binding sites and higher reactivity of the material.

Among the attempts to incorporate Pt-based antitumoral agents, Betsiou et al. evaluated the potential of HAp as a drug delivery system of oxaliplatin, a Pt-salt of the diaminocyclohexane Pt family [69]. The results were compared with the loading and release values of gemcitabine hydrochloride, a difluoro analog of deoxycytidine that inhibits DNA synthesis by replacing cytidine during DNA replication [69,70]. Significant differences in the release kinetics were already found after 1 h of monitoring. In total, 65% and 55% of gemcitabine and oxaliplatin were respectively released in 24 h [69].

Takeyama et al. designed a carboplatin-HAp complex to treat peritoneal carcinomatosis. They added 0.4 mL of a 10 mg/mL carboplatin solution into HAp-containing vials to facilitate the uptake of the drug by the porous mineral nanoparticles. They later tested the antitumor effect by injecting AH130 rat ascites hepatoma cells intraperitoneally into Donryu rats. These were subsequently treated for 7 days either with intraperitoneal saline solution (group I), intravenous carboplatin (group II), intraperitoneal carboplatin (group III) or intraperitoneal carboplatin-loaded HAp (group IV). The results demonstrated an enhanced antitumoral effect and a reduced nephrotoxicity for the carboplatin-HAp combination. Despite the longer survival rates evidenced in group IV, all rats died of peritoneal carcinomatosis [71].

Benedetti et al. tested the loading efficiency of phosphaplatins in HAp NPs, a process facilitated by the chelating pyrophosphate ligand and the negative net charge of the drug at neutral pH. They determined that the optimum phosphaplatin/HAp ratio for biological applications was 10 µM/250 mg per liter. They noticed that phosphaplatin had a low cytotoxicity in HeLa cell lines, but the presence of HAp increased the cytotoxic effect considerably (IC_50_ at 72 h of 10–100 µM alone when compared to 1 µM when HAp NPs were employed). A similar result was evidenced using the HS-5 and MCF-7 cell lines for a 24–72 h time interval [68].

Functionalization of HAp with bisphosphonate or pyrophosphate has also been tested to increase the Pt-based chemotherapeutic loading and the release from HAp structures. This feature is based on the demonstrated role of bisphosphonates to regulate Pt release due to strong interactions with Ca^2+^ ions. The affinity of Pt/bisphosphonate complexes to the cationic anchoring sites can be potentiated by using bisphosphonates with free aminic groups, as proven by Iafisco et al. They investigated the role of Ca/P ratio on the loading of the Pt-bisphosphonate complexes (i) bis-{ethylenediamineplatinum(II)}-2-amino-1-hydroxyethane-1,1-diyl-bisphosphonate and (ii) the bis-{ethylenediamineplatinum(II)}medronate, observing that apatites with high Ca/P ratios have a great affinity for both complexes, but there was a greater upload and lower release of Pt from the first complex. They also tested the Pt complexes released from the apatite against human cervical (HeLa), colon (LoVo), lung (A549) and osteosarcoma cancer cells (U20S). Results corroborated a greater cytotoxic effect than when unmodified complexes were employed [5].

Nadar et al. synthesized a kiteplatin-pyrophosphate compound and loaded it into HAp NPs. A pH-dependent drug delivery was observed, and the anticancer activity of the system was demonstrated for human breast cancer cells *in vitro* and for an embryonic zebrafish xenograft model *in vivo*. In the latest, the tumor reduction capacity of the composite, comparable to the one observed with free kiteplatin-pyrophosphate, was evidenced 2 days after co-injection with eGFP-labeled breast cancer cells [72]. 

Antitumoral drug loading and delivery can also be benefited by the modification of the HAp morphology. Sun et al. performed a detailed investigation in which methotrexate (MTX) was loaded into laminated, rod-like or spherical HAp nanostructures synthesized by using polyethylene glycol (PEG) as a template. MTX is a folic acid derivative and a competitive inhibitor of dihydrofolate reductase. The enzyme inhibition prevents the reduction of dihydrofolate to tetrahydrofolate, blocking DNA synthesis [73]. For this reason, MTX is used as an antitumoral agent, especially in osteosarcoma treatment. The researchers controlled the morphology of the hybrids by changing the pH values to 9, 11 and 10.5, respectively. A higher drug loading capacity was obtained with the first type of hybrid, while the spherical morphology had the lowest value. A slow release kinetic was also observed from the laminated system, with only 40% of MTX being released in the first 10 h of monitoring with respect to the 70% described from spherical and rod-like NPs. In the three cases, a positive correlation of the cytotoxicity with the drug loading capacity was determined [74].

Finally, other antitumoral agents have been successfully loaded into HAp NPs. That is the case of the DDS of fluoropyrimidine 5-fluorouracil based on HAp developed by Tseng et al. The obtained NPs were capable of inhibiting the cell proliferation of A549 cells [75].

## 6. Modified and Doped HAp as an Antitumoral Drug Carrier

Co-substitutions of HAp with trace elements such as Na^+^, Mg^2+^, Zn^2+^, Sr^2+^, K^+^, F^−^ or Cl^−^ can naturally be found in human bone. For this reason, changes in the physicochemical properties, therapeutic potential and possible applications in cancer therapy of HAp doped with these elements have been evaluated [76]. 

Meshkini et al. demonstrated in 2017 that mesoporous zinc-substituted HAp (zinc ion concentration of 1.2 wt%) could also serve as a DDS for cancer treatment owing to the role of zinc in gene expression, cellular metabolism and cell proliferation [77]. They decided to incorporate MTX into the NPs because it is one of the most administered chemotherapy drugs for osteosarcoma. Additionally, as an analog of folic acid (FA), it endows a tumor-targeted activity due to the overexpression of folate receptors (FR) on the surface of cancer. Mesoporous ZnHAp NPs were covered with Pluronic F127 and then MTX was covalently conjugated to the polymer. Based on MTT assays on primary osteosarcoma cells (Saos-2) and an MTX-resistant human osteosarcoma cell line (RSaos-2/MTX), they concluded that the NPs had a higher antitumor efficacy on both cell lines than the free MTX, while the noncytotoxic effect was evidenced on normal human dermal fibroblast cells. They explained the selective effect of the inhibition of P-glycoproteins caused by F-127 and by the release of Zn^2+^ in an acidic environment [7].

**Figure 8:**
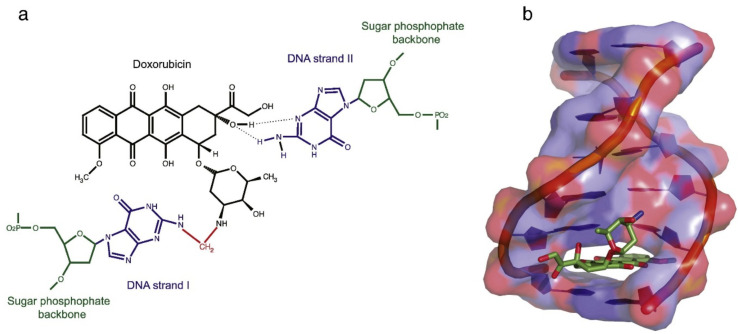
Chemical structure of DOX and mechanisms of action. (**a**) DOX is an anthracycline antibiotic composed of aglycone and an amino-sugar functional group. (**b**) The mechanisms associated to its cytotoxicity can be mainly summarized as: (i) intercalation between DNA base pairs that blocks topoisomerase II activity and prevents DNA replication and RNA transcription, (ii) generation of iron-mediated free radicals that trigger lipid peroxidation and DNA and protein damage. Reproduced from [80]. Copyright © 2013.

Kim et al. also used zinc-doped HAp as a carrier for doxorubicin (DOX). A pH-responsive system was obtained with a higher release rate in acidic conditions and improved bioactivity with respect to systems based on pure HAp [78]. DOX is a widely used pharmaceutical agent for cancer treatment that intercalates between adjacent DNA base pairs, an event that prevents DNA replication and RNA transcription by interfering with the catalytic activity of topoisomerase II (Figure 8) [79,80]. Zinc-doped HAp NPs were loaded with DOX by an adsorption method to treat postoperative bone cancer tissues. It was observed that a Zn substitution degree of around 1 mol% could increase the *c*-axis value, leaving more space to accommodate the drug and increase loading efficiency. The nanoparticles were capable of improving cell attachment, proliferation and osteogenic differentiation when tested on the MG63 osteosarcoma cell line [78].

In recent studies, a positive role of selenium (Se) in skeletal tumor control and prevention of bone cancer metastasis has been discovered [81,82,83]. It has been suggested that the cytosolic release of Se can activate a caspase-dependent apoptosis pathway accompanied by the formation of ROS [81]. Additionally, the combination of Se with Pt-based drugs can help to reduce nephrotoxicity and bone marrow suppression, both effects being attributed to the formation of intermediate Se-Pt complexes [84]. Inspired by these effects, Wang et al. constructed an *in situ* nude mice osteosarcoma model by injecting human SOSP-9607 and tested the antitumoral activity of HAp, 3%Se-HAp and 10%Se-HAp NPs (selenite ions doped at the indicated Se/P mass ratio). The Se ion release from the obtained needle-like NPs was further characterized in a phosphate-buffered saline (PBS) medium. It was found to have a higher release from 3%Se-HAp than from 10%Se-HAp (89.72% and 30.64%, respectively). The lower Se substitution also exhibited the least tumor volume, the slowest tumor growth rate and better biodegradability after 30 days of intertumoral injection into the osteosarcoma [82].

He et al. also investigated the effect of Se substitutions on the structure and antitumoral activity of HAp crystals. HAp and Se-substituted HAp having [Se/(Se + P)] molar ratios of 1%, 5% and 10% were prepared via hydrothermal technique. Se-HAp formulations displayed an oriented growth along the *c*-axis (ribbon-like morphology), and a faster Se release in an acidic medium (i.e., pH 6). Cell viability assays on human osteosarcoma cells SJSA-1 confirmed the cytotoxic effect of Se-HAp on neoplastic cells, obtaining a 55% reduction of viability. Among the tested formulations, 5%Se-HAp also improved the adhesion, spreading and ALP activity of rat calvarial osteoblasts. Hence, these NPs were presented as the best choice for inhibiting tumor growth and showing simultaneously a high bone defect repair [83]. 

In a more recent investigation performed by Barbanente et al., Se-doped HAp NPs with different Se/P ratios were loaded with Pt-pyrophosphate by an absorption process. The release kinetics of both Pt and Se were evaluated. A pH-dependent release of the non-metallic ions was found but not for the drug. In addition, Pt release was affected by the percentage of Se substitution. The cumulative release of Se and Pt after 7 days was ~10 and ~66 wt-%, respectively. From a biological aspect, the compounds were capable of reducing the proliferation of human prostate (PC-3) and human breast cancer cells (MDA-MB-231) without impacting the cell viability of the co-cultured human bone marrow stem cells (hBMSc). The highest cancer cell mortality values with no cytotoxic effect on normal cells were obtained with a Pt/Se ratio of 8 [84].

**Figure 9:**
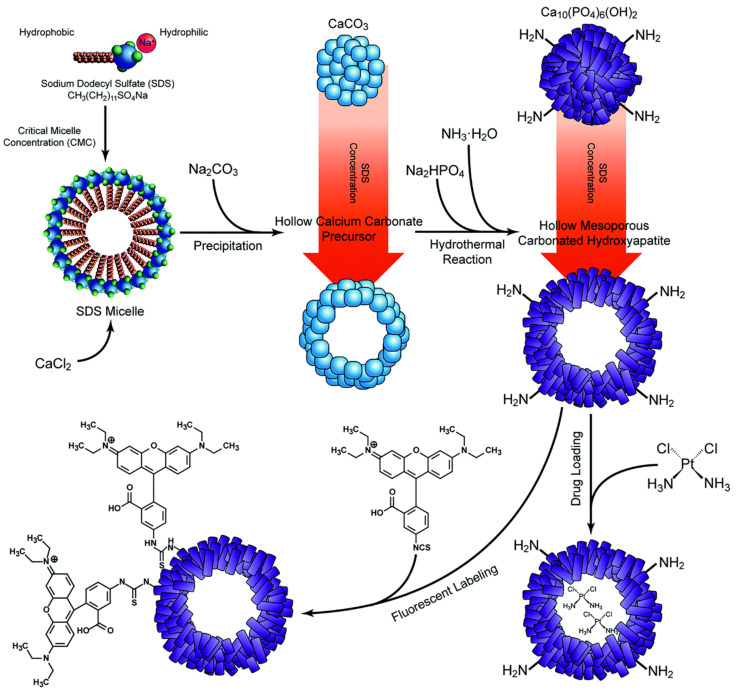
Synthesis of hollow mesoporous carbonated HAp microspheres. Process assisted by SDS for the loading of antitumoral pharmaceutical agents. Image reproduced from [85] with permission from the Royal Society of Chemistry.

To improve the drug loading and release, different hollow HAp microsphere structures have been elaborated. The efficacy of the final drug carrier will depend on the morphology, size and wall thickness of the nanoconstruct. As the listed characteristics can be controlled during synthesis, Qiao et al. proposed the use of different concentrations of sodium dodecyl sulfate (SDS) to assist with the formation of hollow CaCO_3_ precursors. These were later transformed into nanocarbonated HAp crystals via hydrothermal reaction. The resultant hollow mesoporous microspheres were employed to transport cis-diammineplatinum(II) dichloride to human squamous carcinoma cells *in vitro* (Figure 9). The researchers concluded that the optimal morphology, the highest drug encapsulation efficiency and the highest cytotoxicity levels could be achieved when SDS was used at its critical micellar concentration [85].

## 7. Functionalization of HAp and Inclusion into Biopolymer Matrices for Antitumoral Drug Carrier Development 

New antitumoral-drug/HAp formulations are being developed to overcome some deficiencies of the HAp-based nanocarriers, such as the burst drug release, aggregation, the lack of active targeting effect, the low water solubility or the uptake by immune cells that can hinder anticancer activity or increase the off-target effect [5]. To address these issues, HAp NPs are functionalized with different biomolecules andthe nanoformulations already reported are presented in detail in this section. Adittionally, the information has also been summarized in Table 1 and Table 2, located at the end of the section.

**Figure 10:**
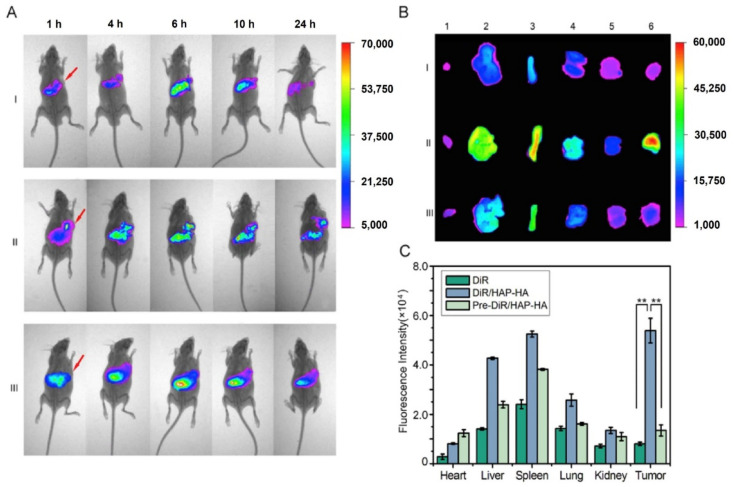
*In vivo* effects of DOX/HAp-HA NPs. In order to evaluate the tumor targeting capability of the NPs, they were labelled with the lipophilic and near-infrared fluorescent cyanine dye DiR. (**A**) Fluorescence images of the *in vivo* experiments carried out on Hep tumor-bearing mice at different time points after the injection of (**I**) free DiR, (**II**) DiR/HAp-HA NPs and (**III**) DiR/HAp-HA NPs with pre-injection of free HA. (**B**) Ex vivo fluorescence imaging of normal and tumor tissues obtained from euthanized Hep cells xenografts bearing mice after 24 h of injection. The numbers are assigned to the heart, the liver, the spleen, the lung, the kidney and the tumor, in corresponding ascendant order. (**C**) Fluorescence intensity analysis (*n = 3*) and *p* < 0.01. Republished from [9] with permission of Elsevier. Copyright © 2016.

Xiong et al. presented a hyaluronic acid (HA) modified HAp system loaded with DOX to provide a hydrophilic coating and a negatively charged surface to the NPs. They observed good stability of the NPs, good drug release rate, biocompatibility and distribution of the particles into both nuclei and mitochondria after 24 h *in vitro*. In detail, the cumulative drug release in PBS after 48 h was higher in pH 5.8 and 4.5 than in neutral conditions (52.3%, 61.4% and 15.6%, respectively). *In vivo* results on mice bearing Hep cell xenografts showed an improved antitumor efficacy attributed to the dual targeting of the system (Figure 10) [9]. 

HA has also been presented as an additional coating for polyethyleneimine (PEI)/HAp NPs. Neutral and negatively charged PEI derivatives have been developed to reduce the cytotoxicity of PEI and allow its use on inorganic NPs to provide colloidal stability. Nevertheless, these modifications decline the phagocytosis ratio and do not guarantee a specific uptake by cancer cells. Kong et al. proposed the attachment of PEI stabilized HAp NPs and the loading of DOX into the resulting system. They demonstrated a pH-responsive release of the drug, a specific affinity of HAp to CD44 overexpressing cells, along with an enhanced cytotoxicity [86].

Natural coatings reported for HAp-based antitumoral materials similarly include polysaccharides such as poly-cyclodextrin for prolonged drug release [87] and chitosan for colloidal stability [88]. In the case of chitosan, Sumathra et al. developed a phosphorylated chitosan/6-gingerol/HAp composite for osteosarcoma treatment based on the antioxidant effect of the pharmacological agent. The investigated system showed an osteogenic potential by inducing ALP activity and expression while suppressing osteosarcoma progression *in vitro* [89]. Xu et al. encapsulated marizomib into chitosan-coated HAp NPs and obtained significantly reduced viability of the A2780 ovarian cancer cells [90]. Chitosan oligosaccharide lactate (ChOLS), a modification of chitosan to improve its water solubility, is another potential molecule for the functionalization of HAp for cancer treatment. Ignjatović et al. encapsulated 3β-hydroxy-16-hydroxymino-androst-5-en-17-one (NP1) and 3β,17β-dihydroxy-16-hydroxymino-androst-5-ene (NP2) into ChOLS-coated nanoHAp, demonstrating a cell-selective toxicity of the pharmaceutical agents against breast cancer cells [91].

Researchers have proposed further contributions from chitosan to the HAp NPs-based systems. For example, Feiz et al. used FA-chitosan as a coating for HAp nanorods to speed adenosine 5′-triphosphate (ATP) release in an acidic pH after the hydrolysis of its glycoside bonds. Moreover, chitosan can transiently open the epithelial tight junctions. Subsequently, it can be anchored to the cell membrane due to its high affinity and lead to an increased internalization of the NPs. These benefits were reflected in the intensified uptake of the system by FR-overexpressing Saos-2 and T47D cells, but not in MCF-7, a cell line with lower FR expression [88].

Cyclodextrin (CD) is a polymer with a hydrophobic character that enables the encapsulation of small hydrophobic molecules [92]. Bischoff et al. showed that the coating of HAp with CD can increase the loading efficiency and release of DOX. In addition, a significant decrease in the survival and growth of MG-63 was described. Nevertheless, the viability of HUVECs under normoxic conditions was decreased to a higher degree. Interestingly, they observed a hypoxia-derived chemoprotective effect on the primary cells that were presented at a lesser degree in MG-63 cells [87].

In an interesting approach, Ramasamy et al. used cyclodextrin-functionalized chitosan to coat strontium-doped and iron/strontium co-doped HAp NPs. The systems were employed as a DDS of DOX. The non-coated mono-doped and co-doped NPs displayed paramagnetic and superparamagnetic properties, respectively. Although a cytotoxic effect was evidenced against lung, cervical, liver and bone cancer cells *in vitro*, an enhanced toxicity was reported on the osteosarcoma MG-63 cells. The nanohybrid was further tested on male and female albino mice, corroborating the antitumoral potential [93].

Another important biomolecule for the development of drug delivery nanoplatforms is collagen. Andronescu et al. loaded cisplatin in collagen/HAp NPs and obtained a cytotoxic and antiproliferative effect in dependence of the cisplatin concentration when tested on osteosarcoma G-292 cell line [94]. Rong et al. loaded collagen/HAp composites with poly(lactide-co-glycolide) microspheres encapsulating DOX (DOX-PLGA-NHAC) [95]. An extended drug release was achieved with the nanocomposite and significant MG-63 inhibition was reported when tested *in vitro*. An *in vivo* model in the rabbit femoral condyle was established to assess the bone repair potential of the scaffold, while a tumor-bearing nude mouse model was considered to evaluate the immune response after scaffold injection. Results suggested no differences between the bone repairing capacity of DOX-PLGA-NHAC and collagen/HAp formulations [95]. A collagen/HAp scaffold was also used by Mondal et al. to develop a dual delivery system of gold (Au) NPs and DOX, considering Au concentrations of 0.1–0.5% [96]. The most recent publication related to collagen/HAp nanocomposites was presented by Matsumoto et al. in 2022. The nanoplatform was used to deliver paclitaxel, which was impregnated on the NPs, to *in vivo* rat models. A local inhibition of osteogenesis was evidenced in the rat femur during the first 8 weeks of the study, with a subsidence at week 12. Treatment with the NPs extended the time to the endpoint from 14 days in the control group to 26.5 days, after which no remaining tumor cells were observed in the femoral bone [97].

Other attempts to modify the surface of HAp-based DDS with biomolecules include the coating with bovine serum albumin (BSA). Li et al. encapsulated DOX into HAp NPs and used BSA as a shielding corona. The protein conferred stability to the compound and augmented its internalization. This was demonstrated by a 1.78-fold increased uptake of the BSA/DOX/HAp NPs by A549 cells with respect to the formulation without BSA. The cytotoxic effect *in vitro* of the BSA-modified NPs was comparable to the free drug and higher than HAp/DOX NPs. The BSA-modified carriers also exhibited a higher internalization *in vivo* and a reduced accumulation in the liver, which suggests a role for BSA in the escape from liver capture. Blood persistence of the NPs was also benefited by the protein coating as a half-life of almost 6 h was described [98].

Surface functionalization with FA can increase the internalization of the NPs due to the up-regulation of folate and CD44 receptors already described in cancer cells [76]. A positive effect of FA on HAp antitumoral activity was demonstrated by Verma et al. They elaborated a FA functionalized gelatin-coated HAp NPs loaded with DOX (DOX-FA-Gel-HAp). They reported a higher uptake by human epidermal carcinoma cells (KB cell line) that overexpressed the FR, compared to the lower intracellular concentration observed in the FR-deficient cell line (human normal hepatic cell line WRL-68) [99]. Functionalized HAp NPs with FA and a gum Arabic coating have also worked as DDS for piperine. A successful delivery of the antitumoral drug to HCT-116 colon cancer cells was found as well as reduced viability values [100]. Izadi et al. also demonstrated the increased antitumoral activity of FA functionalized HAp nanocarriers, but as magnetic properties were conferred to the nanoplatform, the study results will be discussed in detail in Section 8 [101]. 

Doped HAp can similarly be functionalized with biomolecules. This is the case of the selenium-doped HAp (Se-HAp) modified with catechins (Cat), which has been elaborated as a potential DDS in osteosarcoma treatment. The flavonoid was incorporated because of its antioxidant activity, potential to induce apoptosis in osteoclast-like cells and its role as an osteogenic enhancer [102,103]. Even though the resultant structures formed agglomerates, MNNG/HOS cells were capable of internalizing and degrading such NPs. Furthermore, the presence of Cat improved the antitumoral activity of Se-HAp by activating apoptotic mechanisms after the generation of ROS. The normal cells of hBMSc were not affected by the treatment [104].

On the other hand, biomolecules can be incorporated into HAp NPs to coordinate the assembly of the calcium phosphate. As an example, Xiaoyu et al. encapsulated DOX in a pH-sensitive HAp carrier that was formed in a polyglutamic acid-assisted process. The main objectives of its incorporation were to provide complexing sites for Ca^2+^ nucleation, to improve the colloidal stability of the system by steric hindrance, to promote DOX encapsulation and to control the drug liberation. The loading efficiency of the nanohybrid reached 98% in the first 24 h, 20% more than using unloaded HAp. *In vitro* assays revealed the potential of the NPs as Ca^2+^ generators, followed by a decrease in the mitochondrial membrane potential and ATP production. *In vivo* experiments revealed a good antitumoral effect with no evident heart toxicity or hemolysis during the 18 days of study [105]. 

Among the synthetic polymers, polyacrylic acid (PPA) is a polymer that has been grafted onto mesoporous HAp NPs to increase the DOX loading capacity of the system while acting as a pH-responsive switch. The PAA was proposed to bind DOX through electrostatic interactions established with the carboxyl group of the polymer side chain. A physiological pH (7.4) was set during DOX loading to have a protonated antineoplastic agent that could interact with the more deprotonated carboxyl groups of the PAA shell of the NPs. Loading and entrapment efficiency values of 3.3% and 76% were respectively attained. As acidic subcellular environments would lead to the dissociation of the electrostatic interactions, significantly increased release values were obtained in low pHs with respect to those attained in more neutral values (72%, 48% and 15% release after 24 h of monitoring in pH 5, 6.5 and 7.4, respectively). When the cytotoxic activity of the DOX/PAA/mesoporous HAp hybrid was tested using Hep-G2 cells, it found a lower cell viability than the control and an ability to induce apoptosis of cancer cells [6]. 

Polyvinyl alcohol is a semicrystalline synthetic polymer that has recently been used in conjunction with other polymers or inorganic materials to facilitate the loading of both hydrophilic and hydrophobic drugs and control their delivery [106]. Prasad et al. designed two PVA grafted HAp systems for the sequential release of BSA/MTX or gemcitabine (GEM)/MTX. In the first case, they described an initial burst release of both the drug and the protein (∼30%), followed by a sustained release during 13 days of monitoring with final release values of 76% and 88% of BSA and MTX, respectively [107]. In the second case, MTX was conjugated via the hydroxyl group of PVA through an ester linkage and then GEM was physically adsorbed. There was a resultant 20% loading efficiency of MTX and a sustained release with no burst effect of the drug from the system (∼25% release in 10 days). Conversely, 60% of GEM was released during the same time. Cell viability studies on MG-63 cells confirmed the additive cytotoxic effect of the drugs. The system is aimed at targeting osteosarcoma cells with the synergistic activity of both drugs [108]. Other examples of PVA inclusion in HAp nanocomposites for cancer treatment include the DOX/PVA-HAp system developed by Ghosh et al. [109]. 

**Figure 11:**
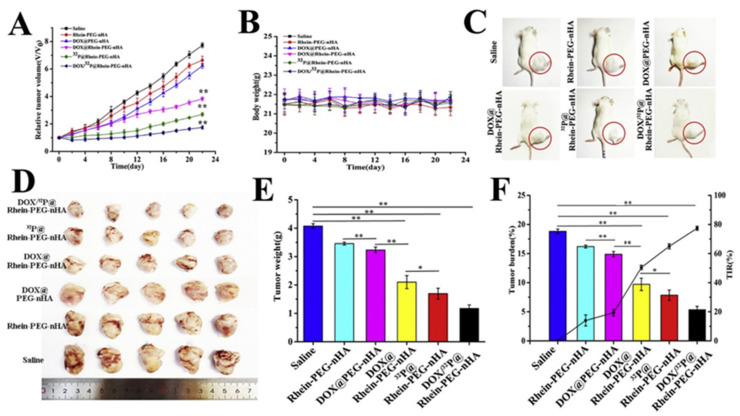
Xenograft tumors collected from mice that shows the reduction of tumoral mass after injection of Rhein-PEG-nano HAp (Rhein-PEG-nHAp): Rhein-PEG-nHAp NPs were synthesized and conjugated with DOX (DOX@Rhein-PEG-nHA), ^32^P (^32^P@Rhein-PEG-nHAp), or both DOX and ^32^P (^32^P/DOX@Rhein-PEG-nHA) for cancer treatment. For *in vivo* studies, Balb/c mice were injected with the NPs to compare the antitumoral effects. Saline, Rhein-PEG-HAp and DOX@nHA-PEG were used as controls. (**A**) Relative tumor volume; (**B**) body weight of Balb/c mice (female, 6–8 weeks); (**C**) representative images of the mice; (**D**) xenograft tumors; (**E**) weight of the collected tumors; (**F**) tumor burden and tumor inhibition rate. In all cases, data represent mean ± SD (*n = 6*) (* *p* < 0.05, ** p < 0.01). Image extracted from [110] with permission from Elsevier. Copyright © 2020.

**Figure 12:**
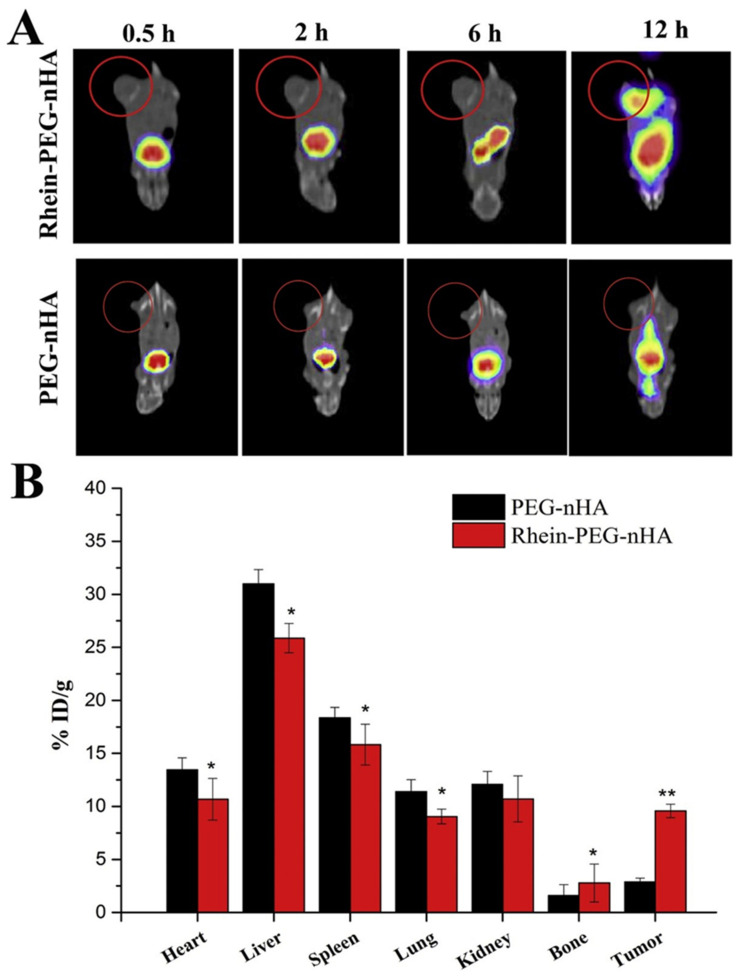
In vivo study of PEG-HAp and Rhein-PEG-HAp NPs for DOX delivery in bone tumor-bearing Balb/c mice model. (**A**) Analysis of the biodistribution of NPs at different time points after injection; (**B**) quantitative analysis of radioactivity uptake of the major organs at 24 h post-injection (*n = 6*) (* *p* < 0.05, ** p < 0.01 vs. PEG-nHA group). Image extracted from [110] with permission from Elsevier. Copyright © 2020.

PEG can be used to link HAp to antineoplastic agents. This is the case of the Rhein-nano HAp conjugated with PEG that Yang et al. presented for a combined chemo-radiation treatment via DOX and Phosphorus-32 (^32^P) delivery [110]. Rhein was selected based on its anti-inflammatory properties and its capacity to inhibit bone resorption by collagenases and metalloproteinases [111]. ^32^P, a β-emitter pharmaceutical, was chosen due to its high biological activity, relatively long half-life and the possibility to use low radioactivity for treatment [112]. The release profile of DOX, as already described in this review, was pH-dependent, but ^32^P displayed good stability. The nanocarrier also showed good bone affinity *in vivo* and a strong growth inhibition of bone metastases of breast cancer was reported when tested in a 4T1 (human breast cancer line) xenograft Balb/c mouse model. Furthermore, DOX/Rhein-PEG-HAp and ^32^P/Rhein-PEG-HAp showed an important decrease in the tumor volume. It is significant that DOX-^32^P/Rhein-PEG-HAp exhibited the highest capacity, which probed a synergistic effect (Figure 11). The distribution of the spherical NPs was also investigated *in vivo* by labeling them with Technetium-99m and tracking them employing SPECT. The results showed a higher accumulation of the Rhein-PEG-HAp NPs in the tumor tissue than observed with PEG-HAp formulations. This process is therefore ascribed to the effective bone targeting of Rhein (Figure 12). Additionally, the researchers also observed a more complete structure of the mice tibias bearing the tumor in the DOX-^32^P/Rhein-PEG-HAp group after 21 days of monitoring. This feature suggests reduced bone resorption and protection of the bone tissue [110]. 

A combined PEG/FA functionalization of HAp has also been developed for the delivery of epirubicin. The NPs, with an average hydrodynamic size of 217.2 ± 14.9 nm, exhibited an increased drug release in acidic pH and an increased uptake by tumoral cells that was attributed to the presence of FA. The inhibition of tumoral growth by the systems was corroborated *in vivo* [113].

PLGA is an aliphatic co-polymer frequently used for DDS due to its *in vivo* biodegradability and biocompatibility. More specially, PLGA can undergo hydrolytic scission, being the resulting lactic or glycolic acid units easily eliminated by the Krebs cycle. As this polymer can increase the mechanical properties and durability of HAp, Ghosh et al. loaded DOX into a PLGA solution to later use it as a coating of calcium phosphate NPs. The obtained particles exerted a cytotoxic effect on MG-63 cells [114]. Promising antitumoral effects against glioma cancer cells were also obtained by Zhang et al. via PLGA/HAp NPs *in vitro* loaded with temozolomide. It was demonstrated that not only the activation of apoptosis but also the decreased expression of αVβ3 integrin, which would translate into a lower invasion potential [115]. 

PLA is a biocompatible polymer often used in the biomedical field with a relatively low degradation rate [116]. HAp NPs loaded with chloramphenicol (CAM) and streptomycin (STR) have been incorporated into electrospun PLA fibers for sustained drug release over a longer period. In the case of CAM, a reduction of approximately 42% in the drug delivery when compared to only CAM-HAp NPs was detected after 20 h of monitoring in PBS medium. The viability of COS-1, VERO and Saos-2 was slightly reduced to 80% [116,117]. For the STR-HAp NPs, FTIR microspectroscopy experiments were performed to evaluate the cytotoxic effect in cancer cells when compared to STR alone. The results showed a significant increase in the intensity of the bands ascribed to the phosphate and glycogen groups and a decrease in the bands assigned to the ribose phosphate backbone and ribose ring in the STR-treated cell group. Moreover, changes between the components of the secondary structure of proteins and a decrease in the lipid content were detected. As for the STR-HAp treated cell group, the separation of the lipid and protein groups was not as clear as the STR group from the control. For this reason, the analysis of the changes in the chemical profile was more difficult to determine. When the STR-HAp NPs were incorporated into PLA fibers, a slower and prolonged drug release was obtained, reaching a 50% at the first 20 h of analysis. In both DDS-PLA scaffold cases, no higher cytotoxicity values were attributable to a slower drug release from the PLA matrices [118]. Other experiments with PLA-HAp were carried out by Lee et al. They encapsulated paclitaxel into PLA-HAp NPs and tested *in vitro* the NPs against murine breast cancer cells, reporting an 80% reduction in cell viability at 48 h [119]. 

Another approach is to combine the strategies so far presented to design more effective antitumoral HAp biobased materials. In this scenario, it is important to evaluate the possible strong binding of the drug with the selected polymers, especially at acid pH values. dos Apostolos et al. reported the production of a mesoporous silica/HAp nanocomposite that incorporated copper (Cu) functionalized with methacrylic acid (MAA) and loaded with MTX. To connect the inorganic network with MAA, they modified the surface by microwave reaction with triethyleneglycol dimethacrylate (TEGDMA). The researchers proposed that the addition of Cu, a hypoxia-mimicking ion, would promote osteogenesis and angiogenesis by activating HIF-1α and VEGF. They demonstrated that the system could effectively encapsulate MTX, but the drug release was faster at pH 7 than at pH 5. Albeit the release profile, cytotoxicity on fibroblast and Saos-2 cells was obtained [73].

Besides the elaboration of drug delivery scaffolds, HAp NPs are considered for regenerative therapy of cancer-affected tissues and simultaneous prevention of tumoral cell reappearance. As an example of this new strategy, Sistanipour et al. chemically functionalized mesoporous HAp with 3-aminopropyltriethoxysilane and then used the stable amide to conjugate Cat. Their research showed promising results for treating bone defects after surgery in osteosarcoma patients. In detail, they demonstrated that the NPs had a higher reactivity toward the hydroxyl and superoxide radicals than free Cat. Results also showed higher adhesion of mesenchymal stem cells, Saos-2 and RSaos-2/Dox cells to glass substrates covered with the bio-construct films. Additionally, the NPs supported the growth of healthy stem cells while decreasing the viability of both cancer cell lines [120]. 

As some surgeries for osteosarcoma resection have led to the utilization of metallic implants to reconstruct bone defects, some researchers have focused their interest on the development of metallic titanium (Ti) implants coated with antitumoral drugs-HAp formulations to render a biocompatible system with a sustained drug release [121]. This is the case of the DOX/chitosan/HAp coated implants presented by Lai et al. They reported an enhanced DOX drug concentration in HAp-coated Ti, attributed to the porosity created by the calcium phosphate on the surface and a slower DOX release [122].

Another example is the vitamin C/HAp-covered implants presented by Sarkar et al. [121]. Mechanisms of vitamin C activity in cancer cells exposed to the uptake of the oxidized form of the molecule via glucose transporters have recently been elucidated. Once inside, the vitamin is reduced again and is accumulated, producing oxidative stress and cell death [123]. Based on these mechanisms, the group explained the increased cell toxicity of MG-63 cells after 3 days of *in vitro* assays. This trend was kept within the 7 days of evaluation, displaying 2.5-fold fewer cells than in the control samples. The controlled release of vitamin C also resulted in firm filopodial attachment and increased proliferation and viability of healthy osteoblast cells on the implant surface [121].

**Table 1:** Functionalization of HAp NPs for cancer treatment.

Surface Modifying Agents	Advantages of the Agent for Functionalization	Antitumoral Drug Loaded	Results
Hyaluronic acid (HA) [9]	Hydrophilic coating Negative surface charge	DOX	***In vitro*** Good stability of NPs Distribution of NPs into nuclei/mitochondria pH-responsive drug release (faster in acidic pH) ***In vivo*** Improved antitumoral efficacy.
HA/PEI [86]	Colloidal stability HA counteracts the phagocytosis ratio decline reported with only PEI	DOX	***In vitro*** pH-responsive drug release Specific affinity of NPs to CD44 overexpressing cells
Poly-cyclodextrin [87]	Prolonged drug release Increases drug loading efficiency Allows the encapsulation of small hydrophobic molecules [92]	DOX	***In vitro*** Higher cytotoxicity on cancer and healthy cells The expression of the tumor suppressor protein p53 is increased
Chitosan [88,90,122]	Colloidal stability Chitosan can transiently open the epithelial tight junctions Can increased NPs internalization by anchoring to cell membrane	ATP [88] Marizomib [90] DOX [122]	***In vitro*** pH-responsive drug release [88,90] Reduced viability of A2780 ovarian cancer Increased cellular uptake and internalization of drug [90], specially by FR-overexpressing cells [88] The NPs were used as titanium implants coating, showing higher DOX concentration on the surface and more controlled drug release [122]
Chitosan oligosaccharide lactate (ChOLS) [91]	Modified chitosan has better water solubility	Steroid derivates	***In vitro*** Increased selectivity towards breast cancer cell lines FACS analysis showed poor uptake of the HAp/ChOSL NPs, so an extracellular drug release was proposed
Phosphorylated chitosan [89]	Modified molecule can induce osteoblast differentiation, covalently attached to different biomolecules and cross-link with Ca^2+^	6-gingerol	***In vitro*** Suppression of osteosarcoma progression Increased ALP activity Increased expression of osteocalcin, osteonectin and transcription factor 2 (Runx2)
Collagen [94,95,96,97]	Better drug loading capacity Increases bone repairing capacity	Cisplatin [94] Poly(lactide-co-glycolide) microspheres encapsulating DOX [95] DOX/gold (Au) NPs (concentrations of 0.1–0.5%) [96] Paclitaxel [97]	***In vitro*** Cytotoxic/antiproliferative effect on osteosarcoma cells. In the case of only HAp/collagen/Au, no cytotoxic effect was described [94,95,96] Extended drug release [95] Incorporation of Au NPs allowed a better cellular attachment, growth and proliferation of bone cells for combined antitumoral/bone regenerative therapy [96] ***In vivo*** No differences were found between DOX-PLGA-NHAC and collagen/HAp NPs [95] Extended the rats survival rate from 14 to 26.5 days while significantly reducing the tumoral cell survival [97]
Bovine serum albumin (BSA) [98]	Stability of the NPs Increases internalization rate	DOX	***In vitro*** 1.78-fold increase in NPs uptake by A549 cells Increased cytotoxic effect ***In vivo*** Higher internalization Reduction in NPs accumulation in the liver Increase in the NPs half-life
Folic Acid (FA)/gelatin [99]	Increases the internalization of NPs in cancer cells due to the up-regulation of folate and CD44 receptors [76]	DOX	***In vitro*** Higher uptake of NPs in FR-overexpressing carcinoma cells vs. FR deficient hepatic cells
FA/gum Arabic [100]	Gum Arabic con prolong the drug release	Piperine	***In vitro*** Successful delivery of the antitumoral agent to colon cancer cells and increased cytotoxicity Cell membrane perforation and disrupted intercellular adhesion were reported
FA/PEG [113]		Epirubicin	***In vitro*** pH-responsive drug release (faster in acidic pH) Increased internalization ***In vivo*** Antitumoral activity demonstrated
PEG/Rhein [110]	Rhein is anti-inflammatory and can inhibit bone resorption by collagenases and metalloproteinase [111]	DOX and Phosphorus-32 (^32^P)	***In vitro*** pH-dependent release of DOX, stable release of ^32^P ***In vivo*** Synergistic effect of DOX and ^32^P Inhibition of bone metastasis of breast cancer and bone resorption Decreased tumor volume
Polyglutamic acid [105]	Offers complexing sites for Ca^2+^ nucleation Improves colloidal stability by steric hindrance Increases drug loading Helps to control drug release	DOX	***In vitro*** Drug loading increase of 20% when compared to HAp alone Decreased mitochondrial membrane potential and ATP production ***In vivo*** Good antitumoral effect No heart toxicity or hemolysis were detected during 18 days of monitoring
Polyacrylic acid (PPA) [6]	Increases drug loading efficiency Behaves as a pH-responsive switch	DOX	***In vitro*** pH-responsive release effect, higher in pH 5 Lower cancer cells viability Induction of tumoral cell apoptosis
Polyvinyl alcohol [107,108,109]	Facilitates the loading of hydrophilic and hydrophobic drugs [106] Controls drug release [106]	MTX and Gemcitabine (GEM) [107,108] DOX [109]	***In vitro*** Release of drug with burst effect in the case of GEM. Additive cytotoxic effect of MTX and GEM was demonstrated [108].
PLGA [114,115]	In vivo biodegradability and biocompatibility Improves mechanical properties and durability of NPs [114]	DOX [114] Temozolomide [115]	***In vitro*** Cytotoxicity of osteosarcoma cells [114] Activation of apoptotic mechanisms, decreased expression of αVβ3 integrin [115]
PLA fibers [116,118] /PLA coating [119]	Biocompatibility, can help to control drug release	Chloramphenicol (CAM) [116] Streptomycin (STR) [118] Paclitaxel [119]	***In vitro*** A 42% reduction of drug release when compared to CAM/HAp NPs alone (for extended drug release). Similar results were observed for STR [116,118] Cell viability of COS-1, VERO and Saos-2 decreased in 20% [116] 80% reduction of cell viability of murine breast cancer cells after 48 h [119]
Aminopropyltriethoxysilane [120]	Facilitates conjugation of the antitumoral agent	1. Catechins (Cat)	***In vitro*** The NPs showed higher reactivity toward hydroxyl and superoxide radicals than free Cat. Increased growth of healthy cells and simultaneous decreased viability of cancer cells

**Table 2:** Functionalization of doped HAp or HAp nanohybrids for cancer treatment.

**Surface Modifying Agents**	**Doping of HAp/Added Agent for HAp Nanohybrid**	**Antitumoral Drug Loaded**	**Results**
Cyclodextrin-functionalized chitosan [93]	Strontium and Strontium/Iron (for paramagnetic and superparamagnetic properties, respectively)	DOX	***In vitro*** Cytotoxic effect in lung, cervical, liver and bone cancer cells ***In vivo*** Antitumoral capacity demonstrated in female albino mice model
Cat [104]	Selenium (for its reported antitumoral activity)	Selenium (Se)	***In vitro*** Albeit the agglomeration of the NPs, they were uptaken by MNNG/HOS cells Cat improved the antitumoral effect of Se Generation of ROS Activation of apoptotic mechanisms
Methacrylic acid (MAA) [73]	Silica/Copper (Cu)	MTX	***In vitro*** Faster drug release at neutral pH Cytotoxicity of fibroblasts and Saos-2 cells
FA/PEG [101]	Fe_3_O_4_	DOX	***In vitro*** Induced hyperthermia generated a decrease in NPs IC_50_ values in Saos-2 Growth inhibition by changing the redox state, caspase activation, apoptosis

## 8. HAp-Based Superparamagnetic NPs for Controlled Hyperthermia in Cancer Treatment

As a new approach for cancer treatment, a combination of magnetic fields and magnetic NPs is being employed to transform energy into force or heat [101]. In detail, when the magnetic NPs are exposed to an alternating magnetic field (AMF), heat is generated through (1) hysteresis losses, (2) frictional losses and (3) Néel and Brownian relaxation losses, affecting the signaling of tumoral cells and inducing apoptosis [124,125]. For biomedical purposes, NPs are required to be superparamagnetic (the ability to be strongly magnetized) under the stimulation of a low-intensity magnetic field. Similarly, they cannot have a remnant magnetization after magnetic field removal because that could possibly cause embolization of capillary vessels due to agglomeration of NPs [126]. Despite the promising aspects of magnetic hyperthermia, it has never been verified that heat treatment can selectively affect cancer cells. In addition, the effectiveness of the magnetic NPs has been reported to be affected but has a lower uptake rate [125]. For this reason, the functionalization of these NPs is one of the latest objectives to revitalize the research on magnetic hyperthermia treatment.

There is a variety of magnetic NPs but the most frequently used in the biomedical field are maghemite (γ-Fe_2_O_3_), hematite (α-Fe_2_O_3_) and magnetite (Fe_3_O_4_) [127]. NPs with these iron oxides are often referred to as surface-engineered superparamagnetic iron oxide NPs (SPIONs) [126]. Albeit their potential for cancer treatment, they exhibit some limitations, such as the oxidation of the NPs and self-aggregation as a result of their high surface energy. These drawbacks can potentiate their segregation by the reticuloendothelial system (RES) and the sudden temperature rise when AMF is applied [124,128]. These problems have been circumvented by the incorporation of such magnetic compounds into inorganic materials such as HAp [129].

Moreover, Zhang et al. have recently presented a model for remote control of apoptosis following the application of a dynamic magnetic field capable of inducing the rotation of SPIONs. The NPs, bound to the lysosomal membrane by the covalently conjugated antibody for lysosomal protein marker LAMP1, tear the organelle membrane during rotation and lead to both the extravasation of lysosomal content and the activation of apoptotic pathways [130].

In this scenario, magnetic properties have been imparted to HAp, providing magnetic drug targeting after AMF [126,128,131,132,133,134,135,136,137,138,139,140,141,142]. Synthesis of magnetic-HAp (mHAp) has been carried out with the following two main approaches: (1) the introduction of magnetic dopant in selected amounts, and (2) the separate synthesis of SPIONs and HAp to subsequently produce a hybrid composite by homogenization methods [139]. For the last process, different techniques have been considered, such as the following: co-precipitation of iron salts and calcium phosphate precursors (under usual conditions of alkaline medium and high temperature) [124], by solvothermal synthesis of magnetic NPs and coating of HAp by wet chemical precipitation techniques [128], hydrothermal-microwave technique [134], hydrothermal synthesis [135], spray-drying technique [136], *in situ* chemical vapor deposition [138], a neutralization method to partially substitute Ca^2+^ by Fe^2+^ and Fe^3+^ while reducing the magnetite secondary phase [126,137,139], mechanochemical reaction [140,141], or byco-precipitation of magnetic NPs and HAp by ultrasonic spray pyrolysis [142]. The variability of the synthesis procedures and iron oxide percentages partially explains the difference in the morphology of mHAp. For example, Murakami et al. [135] characterized rod-shaped NPs while Mondal et al. obtained spherical NPs [128]. The mass percentage of magnetite addition also influences the heat generation by the NPs after magnetic field stimulation. Murakami et al. reported that composites with 30 and 50 magnetite wt-% reached over 43°C within 10 min under the applied magnetic field (i.e., 600 kHz and 3.2 kA/m), but those with a wt-% ≤ 10 could not generate enough heat [135]. Srinivasan et al. analyzed the incorporation of Fe^3+^ into HAp NPs at different sintering temperatures and observed that the presence of FeOOH and α-Fe_2_O_3_ is temperature-dependent. It was also found that the partial stabilization of Fe compounds in carbonated HAp is responsible for an improved induction heating efficiency, achieving saturation magnetization and low coercivity with sintering temperatures of between 400 and 600 °C [143].

The synthesized HAp/SPIONs nanohybrids have been tested *in vitro* with a wide array of cancer cell lines. Pernal et al. evaluated the bioactivity of HAp/SPION nanocomposites by using primary lung and kidney fibroblasts from C57BL/6J mice and human mesenchymal stem cells (hMSCs) as the healthy cell lines, while one osteosarcoma cell line (K7M2-pCl) and two human brain cancer cell lines (U-87 MG, E297) were chosen as cancer cell models. They observed a reduced viability of the two glioblastoma cell lines and an increase in the cell viability of hMSCs when treating them with the nanohybrids. Additionally, the uptake ratio of the NPs by the U-87 MG cells increased in a directly proportional manner to the SPIONs concentration. Studies with a 3D tumor spheroid model revealed a reduced cancer cell migration. The internalized NPs exhibited a perinuclear localization, which indicates the activation of endocytic pathways accompanied by an increase in actin microfilament anisotropy. This feature supports a decreased cell migration [125].

Huang et al. proposed the elaboration of Fe_3_O-HAp-cisplatin NPs for the combination therapy of hyperthermia and chemotherapy. The NPs were elaborated by the microemulsion method, followed by the precipitation of magnetite on the surface. The obtained superparamagnetic NPs were capable of reducing *in vitro* the viability of cancer cells by 24.6%. Interestingly, the nanocarriers continued liberating cisplatin three days after the heat treatment. Activation of apoptotic mechanisms by the mHAp NPs treatment was assessed via Western blot of ERK 2, pERK ½, Bcl-2, cytochrome C and caspase-3. The results suggested the activation of apoptotic mechanisms based on the increased expression of pERK ½, cytochrome C and cleaved caspase-3, combined with a decreased expression of ERK 2 and Bcl-2. The *in vivo* assays, carried out in the BALB/c nude mouse, showed a significant inhibition of tumor growth in the group treated with the cisplatin-loaded mHAp NPs and the application of AMF when compared with the group only treated with mHAp NPs. In the last group, a thermotolerance was exhibited by certain cells within the tumor after 21 days. The researchers attributed this resistance to the activation of heat shock proteins and remarked on the potential of the synergistic effect of hyperthermia and chemotherapy for cancer treatment [132].

In a further effort to elucidate the mechanisms of neoplastic cell death after mHAp-AMF treatment, Yang et al. used cDNA microarrays for whole genome screening of human liver cancer cells Hep-G2 treated with the NPs. Results revealed the downregulation of the DNA-damage related genes ATM, TP53, CDKN1A, GADD45a, GADD45b and GADD45r. The molecular analysis was complemented with Western blotting of proteins of the p38 MAPK pathway, which were found to decrease. As the ATM/ATR pathway is involved in the sensing of DNA damage when cells are exposed to ROS stress, researchers proposed that the mHAp-AMF treatment leads to increased intracellular ROS production. The combined effect with a downregulation of the ROS-response genes led to a significant cell death [133].

The ability to load other antitumoral drugs such as DOX on Fe_3_O-HAp was demonstrated by Iafisco et al. The drug was efficiently released when a low-frequency pulsed electromagnetic field (PEMF) was applied and exerted cytotoxic activity on Saos-2 with results comparable to those of the free drug. Internalization assays showed that DOX was mainly located in the nucleus after 24 h of treatment with the NPs [126].

Magnetic properties have similarly been imparted to mesoporous HAp (MMHAp NPs). These NPs can be synthesized both in a template-dependent [127,129] and in a template-free manner [101]. In the first case, cetyltrimethylammonium bromide (CTAB) and Pluronic F127 have been used [127,129]. For example, Aval et al. developed a mesoporous HAp combined with a superparamagnetic Fe_3_O_4_ core that aimed to deliver DOX to tumoral cells. The tested system showed a good cell inhibitory effect on SKBR3 and T47D breast cancer cell lines [129].

The magnetic NPs can be likewise used to promote osteoblastic activity and bone repair during cancer treatment by the magnetic hyperthermia approach or after tumor excision. Ramya et al. modified Fe^3+^ doped HAp NPs by incorporating Zn to provide antimicrobial properties and inhibit osteoclast differentiation. The NPs synthesized by the ultrasound-assisted technique showed superparamagnetic properties, improved photoluminescence, strong antibacterial activity against Gram-positive bacteria and hemocompatibility. On the other hand, the formation and growth of apatite particles on the surface of pellets made with the material were evidenced after 3 weeks in SBF medium and under controlled conditions. Additional studies are still required to demonstrate the cytotoxic effect on tumoral cells [144].

Progressing in this research line, Li et al. modified PLGA/HAp scaffolds with Fe_3_O_4_ and obtained a porous scaffold with good compatibility. The antitumoral activity of magnetite was proven on MG-63 cells, and a good osteogenic capacity was observed *in vivo* using a rabbit model [145].

Based on these studies, it can be concluded that the effectiveness and selective target activity of mHAp/MMHAp NPs on cancer cells are conditioned by the uptake of the NPs by the cells. Adamiano et al. observed that by allowing the uptake of the NPs by the cancer cells prior to the AMF application, a significant decrease in the cell viability could be obtained (i.e., a higher percentage than observed for the group of cells in surface contact with the NPs coupled to AMF and in a cell line dependent manner) [139]. In order to facilitate the internalization, the magnetic nanoplatforms could be additionally modified. As an example, Izadi et al. functionalized DOX- Fe_3_O_4_-MMHAp nanocarriers with a PEG shell to stabilize them in biological media, followed by a FA conjugation on the surface. Bioactivity was tested on Saos-2 and human embryonic kidney HEK-293 cells. Interestingly, a cytostatic effect of SMF alone was revealed, suggesting a synergistic effect with the magnetic NPs. The hyperthermia induced by the magnetic field resulted in a significant decrease in the NPs IC_50_ values in Saos-2 when compared to HAp alone. Further studies revealed that the mechanisms of growth inhibition included changes in the intracellular redox state, caspase activation and apoptosis [101].

Although mHAp/MMHAp NPs have attracted attention as promising materials for hyperthermia cancer therapy, further studies are still needed to determine if the nanocomposites can produce a better outcome than other calcium phosphates. This was also highlighted by the study of Adamiano et al. They observed that despite a more abundant internalization of the iron-doped hydroxyapatite (FeHAp) NPs than iron oxide nanoparticles coated with amorphous calcium phosphate, the latter induced a higher cytotoxicity of osteosarcoma (K7M2-pCl Neo mouse cells) and glioblastoma cells (E297 human cells). This feature was attributed to a lower lysosomal degradation, blocked particle-particle interactions and superimposition of exchange interactions to Néel relaxation of FeHAp NPs due to a larger size of the HAp-based NPs. These results also emphasize the importance of determining the selective targeting of mHAp against different cancer cell lines and consequently their *in vivo* potential [139].

## 9. HAp as Immunoadjuvant in Cancer Immunotherapy and Gene Delivery System

In vaccine development, the synthesis of a proper adjuvant plays an important role as it enhances the magnitude, quality and longevity of the immune response. Moreover, it helps reduce the amount of antigen required for immunization. Aluminum compounds are an example of adjuvants that are proven to stimulate a Th2 immune response. Nevertheless, the response required for an anti-cancer immunity approach is the activation of Th1 [146]. In the search for better adjuvants for cancer immunotherapy, Wang et al. described in 2016 the potential role of rod-shaped and fluorine-substituted HAp (FHAp). This was based on the observations of an increased uptake of a model antigen and the promotion of immune-related cytokine secretion *in vitro*. Between the two tested formulations, a higher uptake of FHAp-antigen NPs compared to HAp-antigen NPs was observed, representing a better antigen presentation capacity of the first. On the same trend, it was reported a higher interferon-γ (IFN-γ) and interleukin-1β (IL-1β) secretion by bone marrow dendritic cells exposed *in vitro* when mice were injected with FHAp-antigen NPs. In the case of the *in vivo* studies, these results were accompanied by a significant reduction in the tumoral size [146].

The favorable biocompatibility of HAp has also led to its use as a gene delivery system for post-transcriptional gene silencing. Different models of small interfering RNA (siRNAs) delivery based on HAp have been presented in the last 10 years [147,148]. Although HAp NPs can be loaded with different nucleic acids, some surface changes are required to modify the usual weak positive charge to a value sufficiently high to improve both the loading and transfection ability [148].

Liam et al. developed a system for Stat-3 siRNA delivery in order to inhibit the growth of prostate cancer cells. In their study, the *in vivo* analysis revealed a decreased expression level of Stat, PCNA (nuclear proliferation antigen) and CD34 in the C57BL/6 mice treated with the nanocarriers. An immunohistochemical analysis of the tumoral samples showed dispersed chromatin and necrotic tissue and an apoptosis index of 42.56% was reported [147]. 

A strong association between KRAS mutations and the development of metastasis of pancreatic carcinoma has also been reported. To silence KRAS expression, Luo et al. synthesized HAp NPs modified with the PEI cationic polymer to improve the loading efficiency of the KRAS silencing-based siRNA. Only *in vitro* assays were performed on human pancreatic cancer cells (PANC-1, BXPC-3, CFPAC-1) and normal human pancreatic ductal epithelial cells (HPD6-C7). The obtained results indicated a higher siRNA transfection efficiency than a commercial transfection reagent, the knocked expression of the KRAS gene and the downregulation of KRAS protein synthesis. A cytotoxic effect on the cancer cell lines was additionally described. In the case of the normal cell lines, viability values were kept at around 80%, indicating that the reported systems could be further modified to guarantee a higher survival rate of non-tumoral cells [148].

Zhao et al. elaborated a HAp-based system for the co-delivery of a p53 plasmid and candesartan, an angiotensin II type 1 receptor blocker. The nanoconstructs increased the gene transfection rate on MCF-7 cells and inhibited the angiogenesis *in vitro*. The *in vivo* assays performed in nude mice bearing MCF-7 xenografts confirmed the antitumoral activity of the NPs and markers of tumor-associated angiogenesis were decreased [149].

## 10. HAp for Cancer Detection and Cell Imaging

Cancer detection, diagnosis and monitoring of the cellular response to treatment are being approached with noninvasive procedures such as molecular imaging [150]. The visualization and characterization of the biological changes induced by cancer at a molecular-cellular level, in an organ or in the body, can be assessed by using specific imaging probes that provide an analytical signal that is detected more commonly by magnetic resonance imaging (MRI), positron emission tomography (PET) or single photon emission computed tomography (SPECT) [151]. The most frequently used radioisotope is ^99m^Tc due to its good physical and chemical properties, which include a T_1/2_ equal to six and a gamma energy of 140 keV [150]. This radioisotope can be used for labeling a variety of DDS, such as HAp for diagnosis or tracking of the uptake by cancer cells. Among the calcium phosphates, HAp has recently attracted attention as NPs for cell imaging as it has been demonstrated that, when conjugated with organic dyes and lanthanides, they exhibit better fluorescent properties than the amorphous formulations due to rigid confinement of the lanthanide ions in the crystal [5,152]. The advantage of doping HAp with lanthanides is their lower cytotoxicity and photostability. Kataoka et al. proved that the HAp-Eu(III) nanohybrid can be further functionalized with a folic acid derivative (folate N-hydroxysuccinimidyl ester or FA-NHS) by the mediation of 3- aminopropyltriethoxysilane and methyltriethoxysilane molecules. The molecular occupancy ratio of the FA derivative controlled the changes in the photofunctions of the NPs, obtaining the highest quantum efficiency when that ratio represented 3–5%. This allowed for a fast bioimaging of HeLa cells after 1 h of injecting the nanohybrids. Those results were attributable to an intense luminescence from the f-f transition of the Eu^3+^ ions and to the charge transfer between EuTH and FA-NHS [153].

**Figure 13:**
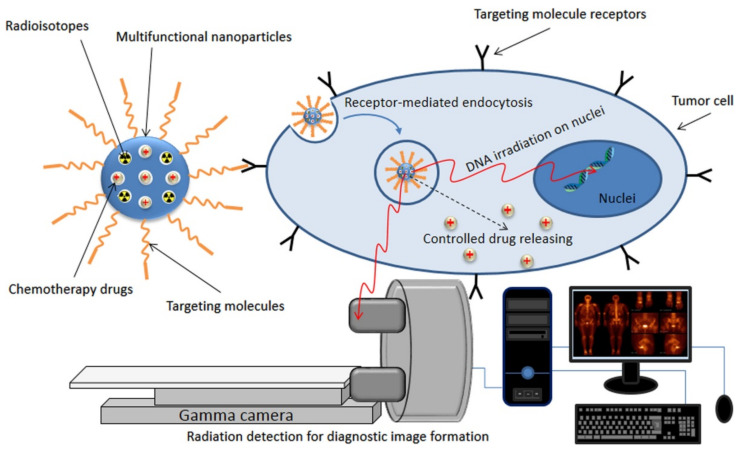
Scheme of a theranostic system. Image reproduced with permission from [154].

An interesting approach is the combination of chemotherapeutics’ loading and radioisotope labeling of HAp for cancer treatment and imaging, also known as theranostics (Figure 13) [124,154]. For example, Shamsi et al. incorporated 2-deoxy-D-glucose and DOX into a ^99m^Tc-labeled mHAp, enhancing the chemotherapeutics cytotoxicity on breast cancer cells [150]. Concisely, the tested nanoplatform showed a reduced cytotoxic effect on slowly growing MCF-7 human cells and significant growth inhibition of rapidly dividing MDA-MB-231 and MC4-L2 (39.8% ± 2.89 and 55.4% ± 3.08 for the human and Balb/c mouse breast cancer cells, respectively). Uptake results showed a 44.77% increase in the internalization of the complex [150].

A recent study also aimed to elaborate a HAp/tenorite with FA to be used as an agent in PET and for the treatment of tumoral cells. Part of the synthesis process involved the activation of copper via neutron flux to produce ^64^Cu inside the HAp matrix, which is a positron and beta radiation emitter. Albeit the promising results, these NPs have not been tested *in vitro* or *in vivo* to further elucidate their potential translational application [154].

SPIONs can also be used as MRI contrast agents for clinical diagnosis. Beeran et al. evaluated *in vitro* the potential of iron oxide-embedded HAp for controlled hyperthermia treatment and MRI contrast efficiency with concentrations of 0.05 mM to 0.25 mM of the NPs on HeLa cells. The signal intensity of T_2_ weighted MRI showed a significant reduction with increasing concentrations. Considering that the contrast enhancement is based on proton relaxation in reference to the local magnetic field and that it occurs on spin-lattice relaxation (T_1_) and spin-spin relaxation (T_2_), the obtained results were attributable to a larger fluctuation in relaxation induced by the NPs. The results indicated a plausible application of the magnetic NPs as negative contrast enhancers in MRI [124]. Gharehaghaji et al. PEGylated mHAp NPs loaded with curcumin to use them as T_2_-weighted MRI agents and DDS. The reported drug loading capacity was 1.9 mg/g and the r2 relaxivity was measured as 120 mM^−1^S^−1^. Antitumoral activity of the system was proven on A549 human alveolar adenocarcinoma cells and MCF-7 breast cancer cells [155].

So far, it can be concluded that doping of HAp NPs with rare-earth ions (e.g., Tb^3+^ or Eu^3+^) can endow the calcium phosphate with luminescence and magnetic properties. Nevertheless, the hydrophobic nature of the final composite can impede its biomedical application. To overcome this problem, recovering the NPs with synthetic amphiphilic polymers is a recent approach to improve water dispersion. Heng et al. presented lanthanide ion (Ln^3+^) doped HAp NPs modified with a combination of surface ligand exchange reaction and reversible-addition fragmentation chain transfer (RAFT) polymerization. In the first reaction, adenosine 5′-monophosphate disodium salt (AMP) displaced the hydrophobic oleic acid on the surface of HAp. Later, the hydroxyl groups of AMP reacted with the carboxyl group of the chain transfer agent. In the second reaction, 2-methacryloyloxyethyl phosphorylcholine (MPC) and itaconic acid (IA) were used as monomers. They demonstrated that the poly(IA-MPC) not only provided a high water dispersibility, but also a large number of carboxyl groups that could coordinate with anticancer agents such as cisplatin in order to elaborate NPs for cell imaging and treatment. Biological assays on A549 cells showed the NPs uptake. Moreover, no cytotoxic effect was obtained with the HAp:Ln-AMP-poly(IA-MPC) formulations, while the cisplatin-loaded NPs decreased the cell viability with a similar effect as cisplatin alone [156].

A HAp-based fluorescent nanosystem has been recently presented for cell imaging and DOX delivery. In this case, nanorods of HAp were functionalized with riboflavin sodium phosphate (HE) for the fluorescent properties, and subsequently, polyethyleneimine (PEI) was attached by electrostatic attraction. NIH-3T3 fibroblasts were treated with the nanocomposites and a fast uptake was reported, allowing correct cell imaging manifested with green fluorescence [157].

## 11. Conclusions

The development of cancer drug delivery systems based on HAp has become a matter of interest in the biomedical and pharmaceutical fields due to its biocompatibility, biodegradability and tailorable properties that allow its fast modification to improve drug loading and controlled release. In addition, HAp NPs can be functionalized in order to obtain a targeted uptake in tumoral cells and to help the NPs escape RES recognition. New studies also highlight the possible antitumoral role of HAp as its lysosomal degradation after internalization can generate high concentrations of cytosolic calcium and trigger mitochondrial-associated apoptotic mechanisms. Based on these properties, not only HAp drug nanocarriers have been designed, but also nanosystems for magnetic hyperthermia, gene delivery systems, adjuvants for cancer immunotherapy and cell imaging have been investigated, mostly *in vitro*. Despite the promising results, there is a lack of *in vivo* studies that could confirm the molecular mechanisms and biological effects produced by HAp NPs for cancer treatment. The present review highlights the need to further develop this research objective into translational medicine.

## Data Availability

Not applicable.
